# Continuous, label-free, 96-well-based determination of cell migration using confluence measurement

**DOI:** 10.1080/19336918.2018.1526612

**Published:** 2018-10-08

**Authors:** Christian Mayr, Marlena Beyreis, Heidemarie Dobias, Martin Gaisberger, Julia Fuchs, Martin Pichler, Markus Ritter, Martin Jakab, Katharina Helm, Daniel Neureiter, Tobias Kiesslich

**Affiliations:** a Institute of Physiology and Pathophysiology, Laboratory for Tumour Biology and Experimental Therapies (TREAT), Paracelsus Medical University Salzburg, Salzburg, Austria; b Department of Internal Medicine I, Paracelsus Medical University/Salzburger Landeskliniken (SALK), Salzburg, Austria; c Institute of Physiology and Pathophysiology, Laboratory of Functional and Molecular Membrane Physiology (FMMP), Paracelsus Medical University Salzburg, Salzburg, Austria; d Gastein Research Institute, Institute of Physiology and Pathophysiology, Paracelsus Medical University Salzburg, Salzburg, Austria; e Ludwig Boltzmann Institute for Arthritis and Rehabilitation, Institute of Physiology and Pathophysiology, Paracelsus Medical University Salzburg, Salzburg, Austria; f Division of Oncology, Department of Internal Medicine, Medical University Graz, Graz, Austria; g Department of Experimental Therapeutics, The UT MD Anderson Cancer Center, Houston / TX, USA; h Institute of Pathology, Paracelsus Medical University/Salzburger Landeskliniken (SALK), Salzburg, Austria; i Cancer Cluster Salzburg, Salzburg, Austria

**Keywords:** Migration, gap-closure assay, confluence measurement, non-endpoint, high-throughput, continuous measurement, cytochalasin D, ouabain, epidermal growth factor, A549 human lung carcinoma cells

## Abstract

Cellular migration is essential in diverse physiological and pathophysiological processes. Here, we present a protocol for quantitative analysis of migration using confluence detection allowing continuous, non-endpoint measurement with minimal hands-on time under cell incubator conditions. Applicability was tested using substances which enhance (EGF) or inhibit (cytochalasin D, ouabain) migration. Using a gap-closure assay we demonstrate that automated confluence detection monitors cellular migration in the 96-well microplate format. Quantification by % confluence, % cell free-area or % confluence in cell-free area against time, allows detailed analysis of cellular migration. The study describes a practicable approach for continuous, non-endpoint measurement of migration in 96-well microplates and for detailed data analysis, which allows for medium/high-throughput analysis of cellular migration *in vitro*.

## Introduction

Cellular migration is fundamental in physiological processes such as embryonic development, tissue remodeling, repair, wound healing and immune response. Migration of cells is also highly important in disrupted pathological situations – especially in tumor biology as invasion and migration of tumor cells are prerequisites for formation of secondary tumors (metastasis) [1–4]. Migration *in vitro* can be defined as cellular movement in a two-dimensional system such as basal membranes or plastic surfaces. Basic characteristics of migrating cells include massive rearrangement of the cytoskeleton, changes in the cellular shape as well as a defined polarity of the cell with a leading edge at the front and a trailing edge at the back part of the cell [3,5,6]. In principle, one can distinguish migration of single cells and so-called sheet migration: in sheet migration, cells retain their cell-cell junctions and move as a collective [3,7]. The formation of secondary tumors is centrally dependent on cellular migration and has high clinical significance, since most cancer-related deaths are caused by metastatic spread [8]. However, tumor cells do not only possess the ability to migrate, but also to invade, meaning they are able to degrade the surrounding extracellular matrix and move through three-dimensional tissue [8]. Although migration and invasion are functionally connected processes in this context, the ability to migrate is a necessity for cells to invade, as non-invasive cells can still migrate, whereas non-migratory cells are not able to invade [3]. Investigation of (two-dimensional) cellular migration is therefore of high interest especially in cancer research.

There are several commercially available migration assays that measure the migratory potential of cells and/or effect of substances or treatments on cellular migration. Despite the fact that these assays cannot entirely mimic the complex process of migration in a (patho-) physiological context, they are popular model systems and widely used in diverse experimental setups (see [3,7] for detailed reviews on migration and invasion assays). Wound healing, i.e. sheet migration movement of cells on a two-dimensional surface to close a cell-free area, is investigated in commonly used assays and protocols and represents a surrogate for the migration potential of cells [9–12]. Applying these assays, a cell-free area is produced by either ‘wounding’ the cellular layer (scratch assay) or by preventing the cellular colonialization of a defined area at the time of cell seeding by chemical or physical stoppers (cell exclusion or gap closure assay). The closure of the cell-free area is monitored microscopically to determine the migration behavior of the cells. Although this assay is very popular and easy to setup, it has several disadvantages. The process of scratching can damage and stress cells, especially at the newly formed border and thereby influence their ability to migrate. Moreover, methods for wounding the cell layer (scratching) as well as size and shape of the scratch vary between laboratories and experiments, which limits reproducibility. The cell exclusion assay also aims to measure the ability of cells to migrate into a cell-free area. However, in contrast to the scratch assay, the cell-free area of the cell exclusion assay is defined before cell seeding by a physical stamp or a gel spot on which cells are not able to grow. After defining the pre-migratory time point, the stamp or gel is removed, and migration of cells can be monitored [3,7,13–15].

Monitoring of cellular migration e.g. *via* a microscope is often performed at defined time points (e.g. after 6, 12, 24, or 48 h) for practical reasons. However, continuous monitoring and evaluation of cellular migration would be advantageous for a detailed characterization of the kinetics of migration and for detailed investigation of the effects of substances or treatments. Therefore, we here describe a modified protocol of the cell exclusion assay that uses the confluence detection function of a multimode reader combined with continuous monitoring of cell migration over a time period of 48 hours under cell culture conditions. As established model substances we used the cardiotonic steroid and Na^+^/K^+^-ATPase inhibitor ouabain ([16–18]) as well as the actin-affecting cytochalasin D [19] as migration inhibitors and the epidermal growth factor (EGF) as a migration enhancer ([16,20]) of A549 human lung carcinoma cells [16,21,22], respectively, to demonstrate the applicability of this protocol.

## Results

**Figure 1.**
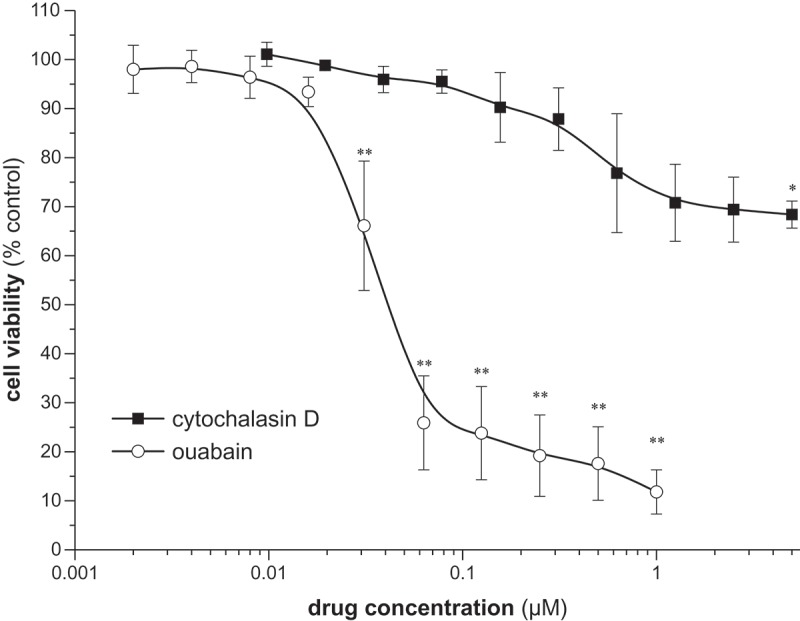
Concentration-dependent effect of ouabain and cytochalasin D on viability of A549 cells after 48 hours. Dots represent the individual data points. Data are presented as mean value ± sem of n ≥ 3 individual experiments compared to untreated cells. * and ** indicate significant (p < 0.05) or highly significant (p < 0.01) differences, respectively.

As described by Liu et al., low concentrations of the cardiotonic steroid and Na^+^/K^+^-ATPase inhibitor ouabain inhibit migration of A549 cells with minimal cytotoxic effects [16]. We therefore treated A549 cells with different ouabain concentrations (dilution series range: 2–1,000 nM) for 48 hours to determine the highest possible concentration that displays only minimal cytotoxic effects. As shown in Figure 1, ouabain concentrations ≥0.06 µM resulted in massive reduction of viable cells, whereas a concentration of 0.031 µM only slightly reduced the amount of viable A549 cells. Although 0.031 µM ouabain displayed small cytotoxic effects we chose this concentration for our further experiments as we wanted to use the highest possible non-lethal concentration to guarantee inhibitory effects of ouabain on migration. Moreover, 0.031 µM are comparable to migration-inhibitory ouabain concentrations described in the literature [16,23]. Likewise, we performed a dilution series with cytochalasin D (dilution series range 0.01–5.00 µM) over 48 hours to determine the highest possible concentration displaying only minimal cytotoxic effects (Figure 1). Based on the results of these experiments, we chose 0.3 µM cytochalasin D for subsequent experiments, as this concentration only showed minimal cytotoxicity (mean survival of 87.9 % related to untreated cells) and is comparable to migration-inhibitory concentrations described by others [19]. For EGF, we used 50 ng/ml as described by Liu et al. [16].

**Figure 2.**
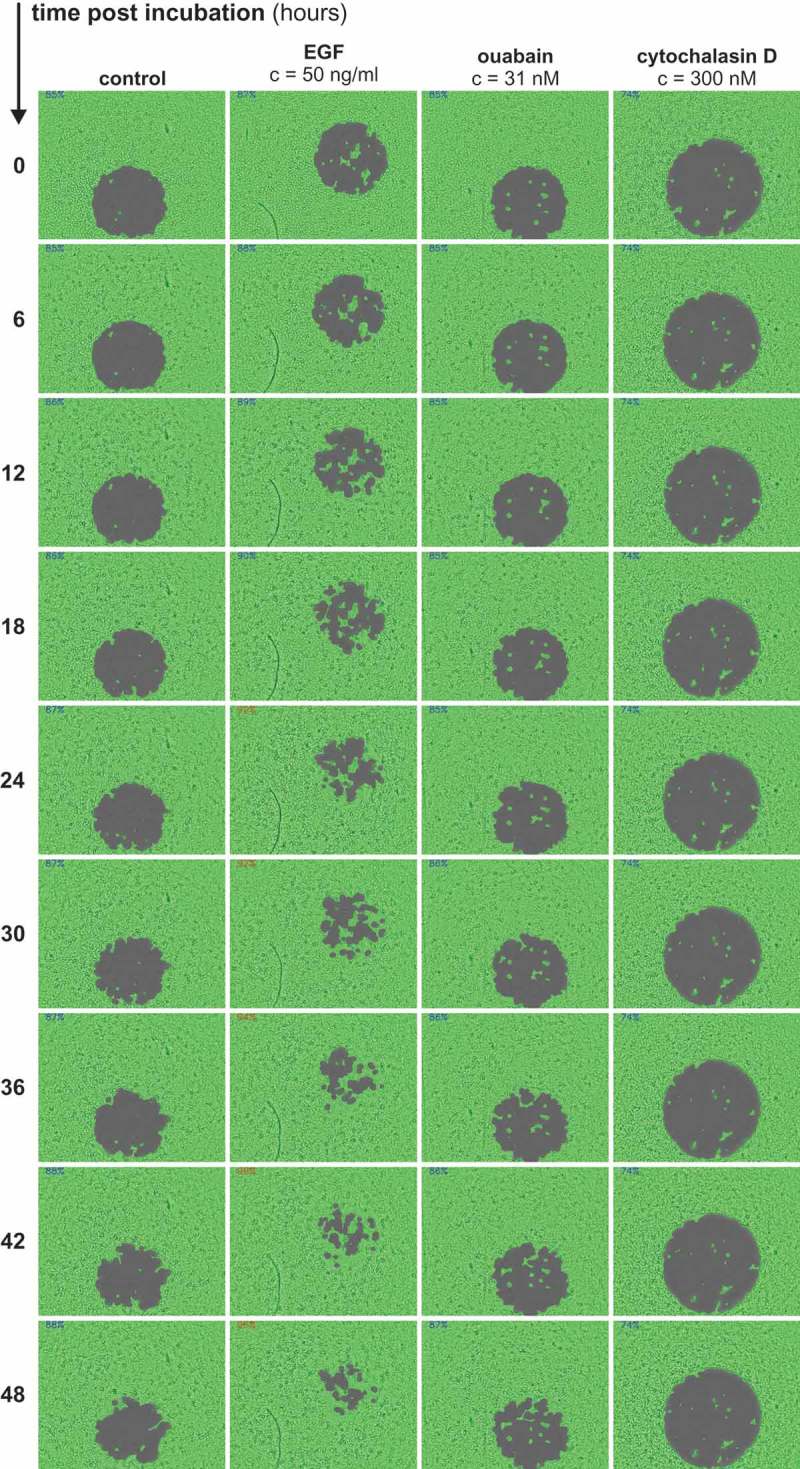
Measurement and quantification of cellular migration using confluence detection. Exemplary series of a non-endpoint and continuous measurement of migration of A549 cells using a gap closure assay (96-well format). Migration was measured using confluence detection under cell culture conditions. Areas detected as confluent are highlighted in green. The same well for untreated control cells, EGF-treated cells, ouabain-treated cells and cytochalasin D-treated cells after 0, 6, 12, 18, 24, 30, 36, 42, and 48 h is shown, respectively. Values in the left upper corner of the images show % confluence of the respective well at the given time point.

To allow continuous and non-endpoint analysis of cell migration, we used the confluence detection mode (imaging) and the environment control functions (CO_2_ and temperature) of a multimode reader. Confluence was measured every 30 minutes for 48 hours at the center of the well where the initially cell-free area (‘spot’) is located. Figure 2 shows an exemplary experimental series for selected time points (0, 6, 12, 18, 24, 30, 36, 42 and 48 h) of untreated, ouabain-, EGF- and cytochalasin D-treated cells, respectively. The confluence detection function was able to reliably detect migrating cells over the course of the experiments (Figure 2) – in case of EGF-treated and cytochalasin D-treated cells, the pro- and anti-migratory effects were obvious by mere visual assessment. Moreover, visual assessment indicates that cells occupy the spot *via* migration and not due to proliferation effects. Morphologically, we observed no cytotoxic effects caused by the protocol of the assay or the substances as indicated by the visual assessment and by the constant confluence in the non-spot area. No effects of the solvent DMSO alone on cellular migration at corresponding concentrations were observed (data not shown).

**Figure 3.**
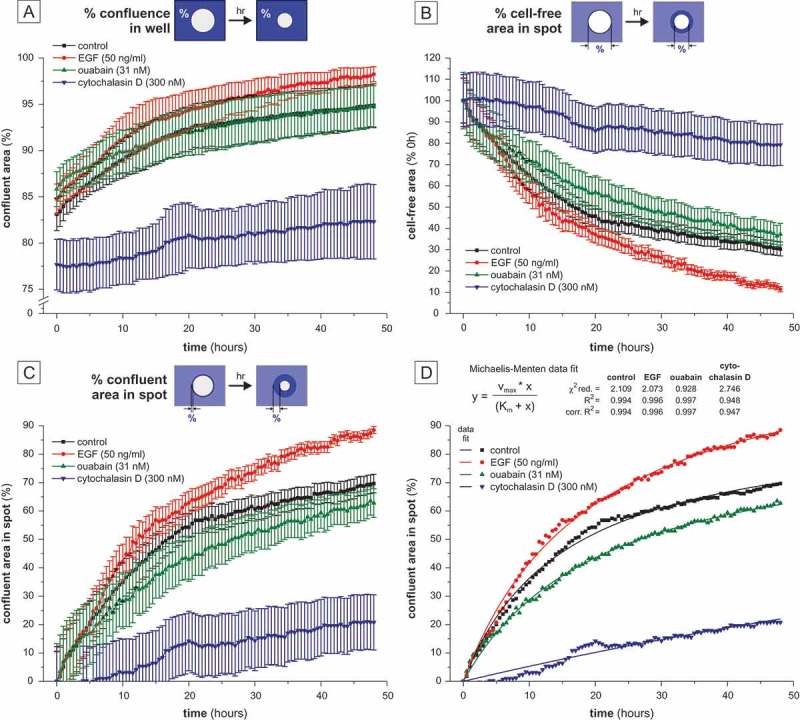
Quantification of cellular migration based on confluence measurement. (a) Quantification of migration as % confluence in the well. (b) Reduction of the cell-free area over time related to the respective 0 h value. (c) Quantification of the confluence in the cell-free area (spot) as *de facto* measurement of cellular migration. (d) Michealis-Menten model of graph C. Dots represent the individual data points. Confluence data displayed in the graphs are based on mean values ± sem of n ≥ 3 biological replicates (each containing at least 3 technical replicates).

As shown in Figure 3(a), a first approach to quantification is based on assessment of the % confluence as provided by the multimode reader’s confluence detection function. However, as the confluence per field of view starts at a rather high value (e.g. for control, starting confluence at 0 h: 85%, confluence at 48 h: 94%) the absolute changes in % confluence were small. Therefore, for subsequent analysis, we changed the calculation approach to relative values only considering the area of the central spot. Due to the settings described in the methods and material section (i.e. high cell seeding density), the non-confluent area visualized by the confluence detection function is exclusively caused by the cell-exclusion of the assay’s gel and not by other effects (such as non-confluent cellular growth of the cells around the gap/wound). Thus, the cell-free area at 0 hours is set to 100% (Figure 3(b)) and migration was quantified as time-dependent reduction of the cell-free/non confluent area. Here, the positive effect of EGF on migration and the inhibitory effects of cytochalasin D and ouabain were clearly discernable, albeit the effect of ouabain is not as prominent. Specifically, the positive effect of EGF on migration is highest between 5 and 15 hours after incubation as indicated by the steeper slope of the curve.

For improved readability, migration can be directly (proportionally) displayed as the confluence in the initially cell-free areas: as shown in Figure 3(c), the spot’s area was defined as 0% confluence at the initial time point, i.e. 0 hours. Using this calculation (% confluence in the spot area *versus* time), the effects of the employed substances on migration can be displayed proportionally: EGF enhances migration of A549 cells meaning that the confluence in the initially cell-free area increases faster compared to untreated cells. Ouabain-treated cells showed a slightly reduced migratory capacity, whereas cells treated with cytochalasin D displayed a clear reduction in cellular migration. Non-linear curve fitting of these data was possible with high (control cells, ouabain- and EGF-treated cells) or good (cytochalasin D-treated cells) quality using a Michaelis-Menten fit function (Figure 3(d)).

## Discussion

Although 2-dimensional measurement of migration does not accurately mimic the physiological 3-dimensional environment, it is an important and widely used surrogate for interpretation of cellular migration [24,25]. In the current manuscript we have described that automated confluence measurement is an efficient tool to measure 2-dimensional cellular migration using the gap closure assay. Due to the functional parameters of the confluence measurement, this protocol is most suitable for the 96-well format. The following thoughts have to be taken into account to gather proper results using our described approach. First, for correct measurement it is important that the cell-free area is completely within the measurement field of view of the confluence detection function, which is feasible using commercially available 96-well microplate format gap closure plates. However, probably due to variations in the manufacturing process, we noticed that the cell-free area spots were not always located at the center of the well, making interpretation difficult. In fact, several experimental series had to be excluded from our data analysis due to extreme variations in the spots’ positions. We therefore recommend to include sufficient technical replicates and, if technical feasible, to extend the segment of the well at which confluence is measured to correct for these variations in the spots’ positions. Second, to perform the analysis described in the current manuscript, it is mandatory that cells are completely confluent around the cell-free area, which can be easily achieved in the 96-well microplate format by using sufficient cell densities. Importantly, visual evaluation of cell confluence around the cell-free area and cell health using a microscope is mandatory before starting the experiment to ensure reliable results. Nevertheless, variations between biological replicates are possible. Although the trends within each individual experiment were always observable (e.g. migration-promoting effect of EGF; migration-inhibitory effect of ouabain and cytochalasin D), we found that the magnitude of these effects varied between the biological replicates. From a technical point of view, confluence measurement including sufficient biological replicates was able to resolve the pro- and anti-migratory effects of the employed model substances despite the fact that their absolute effects varied between the biological replicates. Lastly, while the described protocol requires no additional hands-on time after starting the experiment since the measurement is performed continuously within the instrument, the microplate reader must be equipped with the option to control the CO_2_ concentration, temperature and prevent evaporation form the plate to ensure correct environmental conditions.

Our protocol offers several advantages compared to standard protocols without continuous confluence measurement [3,7,13]. First and foremost, measurement of confluence in a multimode reader equipped with temperature and gas control allows non-endpoint and continuous monitoring of cellular migration without any hands-on time after starting the assay. In contrast to conventional endpoint measurements and/or measurements at defined time points, continuous investigation of migration allows a more accurate interpretation of the migratory potential of different cell lines and of the effects of substances or treatments [25]. Moreover, this protocol allows automated and medium/high-throughput monitoring of migration due to described software settings without the need to move the plate from the cell culture incubator to the multimode reader. In addition, the protocol is flexible in the sense that if adapted to individual experimental setup and used wells, timing of the confluence measurements can be adjusted. For example, in the current setup we used up to 28 wells per experimental series and measured the confluence in each well every 30 minutes. Dependent on the number of used wells per experiment (and the associated amount of time required for confluence detection) one can use a more close- or broad-meshed time schedule for confluence measurement. Furthermore, the 96-well format allows high-throughput analysis including appropriate controls and technical replicates and also minimizes the amount of cells and chemicals required compared to other assays such as the scratch assay, which is often performed in tissue culture dishes [26]. Another advantage of our protocol is that the detailed and quantitative investigation of migration data obtained by confluence measurement highlights several ways of data evaluation. As demonstrated, besides analysis of the confluence per well/per field of view itself, one can also quantify the reduction of the (initially) cell-free area and the increase of the confluence in this area (gap) in order to directly quantify cellular migration into the gap. Dependent on the results, a Michaelis-Menten data fit with high quality can be performed as shown for our results. For biological questions, the formula of the data fit can be used to quantify migratory behavior under defined circumstances, e.g. one could calculate the required time at which 50% of the cell-free spots are populated by migrated cells to provide a single quantitative measure of cell migration under different experimental conditions. This is especially interesting when used as a semi-high throughput screening approach for substances and/or treatments. Such detailed analysis of effects of substances or treatments on cellular migration based on continuous confluence measurements therefore allows a more detailed and accurate analysis and consequently allows additional scientific statements.

In conclusion, the current manuscript describes a new and expanded protocol for measurement of cell migration using microplate-based imaging and confluence determination enabling non-endpoint, medium/high-throughput and continuous data generation. Combined with the described options for data analysis, the protocol represents a versatile tool for medium/high-throughput studies of cellular migration and for evaluation of the pro-/anti-migratory effects of diverse substances and treatments.

## Materials and methods

### Cell culture and substances

A549 lung carcinoma cell lines [27] were cultured in RPMI 1640 medium (Thermo Fisher Scientific, FG1215) supplemented with 10% (v/v) fetal bovine serum (FBS; Biochrom, BC-S0615), L-glutamine (Gibco, 25,030,081) and antibiotic-antimycotic (ABAM, Sigma Aldrich, A5955). Cytochalasin D was purchased at ThermoFisher Scientific (PHZ1063), dissolved in dimethylsulfoxid (DMSO, Sigma Aldrich, D4540) at a stock concentration of 5 mM, aliquoted and stored at −20°C. Ouabain was purchased at Sigma Aldrich (O3125), dissolved in DMSO at a stock concentration of 20 mM, aliquoted and stored at −20°C. Epidermal growth factor (EGF) was purchased at Thermo Fisher Scientific (PHG0311L) as a stock solution of 1 mg/ml and stored at −20°C. Resazurin was obtained from Sigma Aldrich (R7017), dissolved in Dulbecco’s Phosphate Buffer Saline (DPBS, Biochrom, L1825) and stored at −20°C.

### Cytochalasin and ouabain cytotoxicity

Cells were seeded in 96-well microplates at a concentration of 2 × 10^5^ cells/ml corresponding to 2 × 10^4^ cells per 100 µl and well. A dilution series of cytochalasin D (range 0.01–5.00 µM) or ouabain (range: 2–1,000 nM) was applied for 48 hours. Serum-free media (sfRPMI) was used to avoid potential interactions of serum components with cytochalasin D/ouabain. Quantification of viable cells was done using the resazurin assay and a Spark® multimode reader (Tecan). Viability was related to untreated cells (incubated with sfRPMI only).

### Gap closure assay

The Radius^TM^ 96-Well Cell Migration Assay (Cell Biolabs, CBA-126) was used according to the manufacturer’s instructions. For all experiments, sfRPMI was used to avoid cellular proliferation during measurement [25]. In brief, A549 cells were seeded at high density (6×10^5^ cells/ml; 6 × 10^4^ cells per 100 µl and well) overnight in the Radius^TM^ 96-Well Cell Migration Assay plate. After washing, the gel was removed and cytochalasin D (0.3 µM), ouabain (0.031 µM) or EGF (50 ng/ml) were added in sfRPMI. For continuous monitoring, the plate was placed in the Spark multimode reader for 48 hours under standard cell culture conditions (37°C, 5% CO_2_) using the temperature and gas control of the reader. To avoid evaporation, the plate was placed in a ‘large humidity cassette’ which includes excess water in a groove surrounding the microplate (Tecan). Confluence measurement of each well was performed every 30 minutes using the confluence measurement function in the center of the well using a kinetic loop (96 measurements, measurement every 30 minutes). Before each experiment, the position of the cell-free area as well as the density and health of the cells were verified using a phase-contrast microscope (Motic) and the microplate reader’s Live-Viewer function to guarantee that confluence measurement in the center of the well is suitable to cover the gel spot and most of the surrounding area.

### Statistics

All data points represent mean values of four individual biological replicates ± standard error of mean (sem) or Gaussian error propagation were applicable. For confluence measurements, the individual biological replicates included at least three technical replicates. Paired Student’s t-test was used for calculation of significant differences between control and ouabain/cytochalasin D viability, respectively. Statistical results were considered significant (*) or highly significant (**) at p < 0.05 and p < 0.01, respectively. Calculations and diagrams were performed with OriginPro 2017 (OriginLab).
